# First Nationwide Monitoring Program for the Detection of Potentially Invasive Mosquito Species in Austria

**DOI:** 10.3390/insects13030276

**Published:** 2022-03-10

**Authors:** Karin Bakran-Lebl, Stefanie Pree, Thomas Brenner, Eleni Daroglou, Barbara Eigner, Antonia Griesbacher, Johanna Gunczy, Peter Hufnagl, Stefanie Jäger, Hans Jerrentrup, Lisa Klocker, Wolfgang Paill, Jana S. Petermann, Bita Shahi Barogh, Thorsten Schwerte, Carina Suchentrunk, Christian Wieser, Licha N. Wortha, Thomas Zechmeister, David Zezula, Klaus Zimmermann, Carina Zittra, Franz Allerberger, Hans-Peter Fuehrer

**Affiliations:** 1 Institute for Medical Microbiology & Hygiene, AGES—Austrian Agency for Health and Food Safety Ltd., 1096 Vienna, Austria; peter.hufnagl@ages.at (P.H.); franz.allerberger1@gmail.com (F.A.); 2 Institute of Parasitology, Vetmeduni Vienna, 1210 Vienna, Austria; stephanie.pree@gmail.com (S.P.); barbara.eigner@vetmeduni.ac.at (B.E.); bita.shahibarogh@vetmeduni.ac.at (B.S.B.); licha.wortha@vetmeduni.ac.at (L.N.W.); hans-peter.fuehrer@vetmeduni.ac.at (H.-P.F.); 3 GEBL—Gelsenbekaempfung in den Leithaauen, 2452 Mannersdorf, Austria; thomasbrenner08@gmail.com; 4 Verein Biologische Gelsenregulierung March-Thaya Auen, 2273 Hohenau an der March, Austria; eleni.dar21@gmail.com (E.D.); hans.jerrentrup@gmx.at (H.J.); 5 Data, Statistics & Risk Assessment, AGES—Austrian Agency for Health and Food Safety Ltd., 8010 Graz, Austria; antonia.griesbacher@ages.at; 6 Universalmuseum Joanneum, Studienzentrum Naturkunde, 8045 Graz, Austria; johanna.gunczy@museum-joanneum.at (J.G.); wolfgang.paill@museum-joanneum.at (W.P.); 7 Department of Zoology, University of Innsbruck, 6020 Innsbruck, Austria; stefanie_jaeger@live.de (S.J.); thorsten.schwerte@uibk.ac.at (T.S.); 8 Inatura, 6850 Dornbirn, Austria; klocker@naturpark-nagelfluhkette.eu (L.K.); klaus.zimmermann@inatura.at (K.Z.); 9 Environment and Biodiversity, University of Salzburg, 5020 Salzburg, Austria; jana.petermann@plus.ac.at (J.S.P.); davidzezula@gmx.at (D.Z.); 10 Biological Station Lake Neusiedl, 7142 Illmitz, Austria; carina.suchentrunk@bgld.gv.at (C.S.); thomas.zechmeister@bgld.gv.at (T.Z.); 11 Landesmuseum Kärnten, 9020 Klagenfurt, Austria; christian.wieser@landesmuseum.ktn.gv.at; 12 Department of Functional and Evolutionary Ecology, University of Vienna, 1030 Vienna, Austria; carina.zittra@univie.ac.at

**Keywords:** *Aedes albopictus*, *Aedes japonicus*, *Aedes geniculatus*, Asian tiger mosquito, Asian bush mosquito, invasive species, ovitraps, vectors

## Abstract

**Simple Summary:**

In the last years several alien mosquito species have been introduced into Austria. Those species pose a threat, as they—especially the Asian tiger mosquito (*Aedes albopictus*)—can transmit many pathogens. The aim of this study is a nationwide overview on the situation of alien mosquitoes in Austria. Using traps representing ideal breeding sites for those mosquitoes, we collected, counted and identified the species of the mosquito eggs laid in the traps. The Asian tiger mosquito was found at two sites, once in Tyrol, where this species has been reported before, and for the first time in the province of Lower Austria. The Asian bush mosquito (*Aedes japonicus*) was widespread and abundant in Austria. Although it was found in all provinces, the Asian bush mosquito was more often found in the South than the North and more eggs were collected in urban/industrial/transport areas than in (mostly) natural areas. Further, more eggs from the Asian bush mosquito were found in samples collected at higher daily mean temperatures, and fewer eggs in samples collected at higher daily maximum wind speeds. The results of this study will help to better understand the risk from alien mosquitoes to human health in Austria and will be useful to show future changes in the distribution of those species.

**Abstract:**

In Austria, only fragmented information on the occurrence of alien and potentially invasive mosquito species exists. The aim of this study is a nationwide overview on the situation of those mosquitoes in Austria. Using a nationwide uniform protocol for the first time, mosquito eggs were sampled with ovitraps at 45 locations in Austria at weekly intervals from May to October 2020. The sampled eggs were counted and the species were identified by genetic analysis. The Asian tiger mosquito *Aedes albopictus* was found at two sites, once in Tyrol, where this species has been reported before, and for the first time in the province of Lower Austria, at a motorway rest stop. The Asian bush mosquito *Aedes japonicus* was widespread in Austria. It was found in all provinces and was the most abundant species in the ovitraps by far. *Aedes japonicus* was more abundant in the South than in the North and more eggs were found in habitats with artificial surfaces than in (semi-) natural areas. Further, the number of *Ae. japonicus* eggs increased with higher ambient temperature and decreased with higher wind speed. The results of this study will contribute to a better estimation of the risk of mosquito-borne disease in Austria and will be a useful baseline for a future documentation of changes in the distribution of those species.

## 1. Introduction

Facilitated by global change, several exotic mosquito species have been introduced into Europe in recent decades [1,2]. The increased transport of goods, as well as an increasing mobility of humans and pets around the globe promotes the unintentional introduction of alien mosquitoes, especially of the *Aedes*-genus, as they can produce eggs resistant to desiccation [3,4]. After their arrival in Europe, populations can become established if suitable climatic conditions exist [1,5]. Urbanization and climate warming are likely to increase their breeding success and survival and thus may expedite the establishment and spread of these species in Europe [6,7,8].

Those introduced alien species can become invasive, if they cause or are likely to cause economic or environmental harm or harm to human health [9]. According to the European Centre for Disease Prevention and Control (ECDC), six species of *Aedes* invasive mosquitoes (AIMs) have been introduced into Europe in the recent past: *Aedes aegypti*, *Ae. albopictus*, *Ae. atropalpus*, *Ae. japonicus*, *Ae. koreicus* and *Ae. triseriatus* [10]. Although they are generally referred to as invasive species, not all of them have been shown to fulfil the definition of invasiveness, as their interaction with the local ecosystems and/or their vector competence remains to be investigated.

Up to date, three AIM species have been recorded in Austria. In 2011, *Ae. japonicus* was detected for the first time in this country, and has ever since been found often and at many locations [11,12,13,14]. One year later, in 2012, *Ae. albopictus* was reported for the first time. In the following years, this species was occasionally found near major motor highways and in urban areas (in the towns Lienz, Kufstein and Innsbruck) in western Austria (Tyrol) [12,13,15]. It remains uncertain whether *Ae. albopictus* has been repeatedly introduced or if it has established populations in those areas. In 2020, this species was documented for the first time in an Austrian metropolitan area, in the city of Vienna [16]. Most recently, a few specimens of *Ae. koreicus* have been found in Austria in 2012, 2017 and 2018 [15,17]. Those AIMs pose a potential public health risk in Austria as they may transmit pathogens, including those that cannot be transmitted by native mosquito species. Especially the establishment of the Asian tiger mosquito (*Ae. albopictus*) poses a risk, as this species is a competent vector for many pathogens such as Dengue-, Chikungunya- and Zika-virus, but also for *Dirofilaria* nematodes [2,18,19,20]. Following the establishment of *Ae. albopictus*, several disease-outbreaks with autochthonous transmissions have already occurred in Southern Europe (e.g., [21,22,23]). The other two AIM species in Austria are also known to transmit pathogens, although most studies were conducted at laboratory conditions (*Ae. japonicus*—e.g., West-Nile virus, *Ae. koreicus*—Japanese encephalitis virus). Generally, *Ae. japonicus* and *Ae. koreicus* are considered less important disease vectors [2,24,25]. The three AIM species already detected in Austria are known to be anthropophilic and, in contrast to most native species, active during daytime. This intensifies the contact rate to humans, which not only makes them nuisance biters, but also increases the risk of disease transmission.

To protect the Austrian public from the risks posed by these AIMs, it is essential to know where and when these alien mosquitoes occur. It is important to detect AIM populations as early as possible (especially *Ae. albopictus* populations), because only then effective countermeasures can be taken to eliminate or at least reduce these mosquito populations [10]. In the past, several monitoring programs have investigated parts of the Austrian mosquito fauna, but with varying levels of effort and using different methods [12,15,26,27]. To obtain comparable results, however, it is important that a unified sample protocol is used. This study aims to obtain for the first time an overview of the geographic and seasonal distribution of AIM species throughout Austria.

## 2. Materials and Methods

### 2.1. Trap Sites and Catch Scheme

Preceding project start, volunteers were recruited to perform the sampling at the mosquito traps. These volunteers included mosquito experts (from academia as well as from mosquito control institutions), zoologists from natural history museums or research facilities, as well as interested non-professionals. All necessary materials, a detailed instruction and forms to record the data during sampling were provided to them. In total, 32 people contributed to the sampling of the eggs, whereby some were responsible for several trap sites whereas at other trap sites the sampling was done by several people.

Sites chosen for monitoring were primarily (but not exclusively) those in urban or suburban areas, as well as locations where alien species might be introduced into the country (e.g., airport, motorway service areas, train stations). A total of 45 sites was selected (for details on the trap sites see Supplementary Materials). Although the sample sites cover all federal provinces, the distribution of the sites was irregular according to the availability of volunteers in a given area. At each site, traps were placed at five positions, however at four sites more traps were used to cover a specific area (e.g., 20 traps at the airport), or fewer traps were placed (1–4 traps at 6 locations).

To prevent the traps from influencing each other, it was specified that they should be 15–100 m apart and assignable to the same habitat. However, these standards could not always be adhered to, as at 7 sites the distance to the nearest trap was 7.6–15 m, and at 16 sites the distance to the nearest trap was 100–1802.0 m for at least one pair of trap positions (for details see Supplementary Materials). Those deviations from the protocol were caused by a low availability of suitable trap locations at a specific site (e.g., airport) or misjudgment of distance by the volunteers. Thus, the actual distance to the nearest positions was on average (± standard deviation) 91.9 ± 203.7 m. Care was taken to choose positions that were undisturbed by people or animals, e.g., by choosing (if possible) positions in private property. The instruction for the volunteers was to choose the positions in such a way that these were shaded and moist (e.g., in bushes) to avoid the risk of drying out and to provide suitable resting places for adult mosquitoes.

For the detection of alien mosquitoes, we used ovitraps, which represent an ideal breeding habitat for container-breeding *Aedes*. These traps make use of the fondness of this genus to lay eggs on a moist substrate (in contrast to other mosquito genera like *Culex*, who lay their eggs on the surface of water bodies). Eggs are laid on a provided oviposition support in the trap, which is collected at regular intervals and checked for the presence of eggs. At each position an ovitrap was set up from the beginning of May (with the exception of 12 sites where the sampling period started in June or later) until the end of October. This observation period was chosen as it corresponds to the period in which alien mosquitoes have been detected in Austria so far (June–September [12,15,26]), plus one month before and after, in order to record the beginning and end of the egg laying season. The traps consisted of 1-L black plastic cups (height: 13.25 cm, diameter (top): 13.23 cm) and were filled with approximately 0.75 mL tap water. A wooden mouth spatula (15 × 1.8 cm), which was fixed to one side of the cup with a stainless-steel clamp, was used as oviposition support. Traps were checked and the oviposition support as well as the water were changed weekly, although longer intervals (up to 36 days; caused by limited availability of personnel at the participating institutions, by vacation or illness) could occur, resulting in an average (± standard deviation) time interval of 8.3 ± 3.8 days between trap checks. The sites in the province Vorarlberg could only be checked on average every 17.7 days. Thus, a larvicide was added to the cups (2.5 mL Gnatrol^®^/L, active ingredient: B.t.i.). The oviposition supports were packed in small zip-lock bags and sent by mail in a padded envelope to the laboratory in Vienna.

### 2.2. Sample Analysis

**Figure 1:**
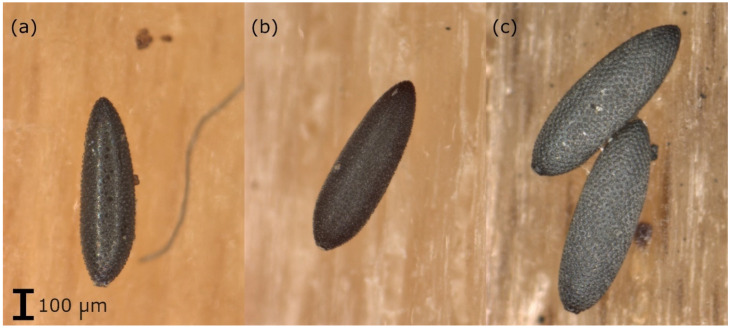
Eggs of (**a**) *Ae. albopictus*: eggs with a shiny black surface, with symmetrically arranged large but narrow tubers in the center of the chorionic cells, (**b**) *Ae. japonicus*: black eggs with a matte surface, with uneven and irregularly arranged tubers and (**c**) *Ae. geniculatus*: eggs with a black surface, with symmetrically arranged flat but broad tubers in the center of the chorionic cells, larger than *Ae. albopictus* or *Ae. japonicus* eggs.

Samples arriving at the laboratory were kept in the refrigerator until analysis (if the analysis was not possible within 5 days, they were stored in the freezer at −20 °C). The oviposition supports were checked for the presence of *Aedes* eggs using a stereo-microscope. The eggs (including those already hatched) were counted and a preliminary morphological species determination of the eggs was carried out based on their surface structure (Figure 1 [28,29]). The eggs were placed in Eppendorf tubes (1.5 mL). If based on the morphological analysis, it was suspected that eggs of different species were on one oviposition support, about 5–10 eggs of each species (if that many were available) were placed in a separate tube. The tubes were stored at −80 °C until genetic analysis.

After homogenization of eggs in a TissueLyser II (Qiagen, Hilden, Germany) with a ceramic bead (2.8 mm Precellys Ceramic Beads, VWR, Darmstadt, Germany) as described previously [12], DNA was isolated either using the Qiagen DNeasy Blood&Tissue kit (Qiagen, Hilden, Germany) or innuPREP DNA Mini Kit (Analytik Jena, Jena, Germany) according to the manufacturer’s instructions. To identify insect species, barcoding was performed within the mitochondrial cytochrome oxidase subunit I (mt COI) gene using the primers LepF1 and LepR1 [30]. PCR products were sequenced at LGC Genomics GmbH, Berlin, Germany. The resulting sequences were compared to sequences available on BOLD Systems (www.boldsystems.org, accessed on 18 January 2022) and GenBank (www.ncbi.nlm.nih.gov/genbank, accessed on 18 January 2022) databases.

### 2.3. Data Analysis

To take different lengths of sampling periods (the number of days an oviposition support was in a trap) into account, data was standardized: The number of eggs on an oviposition support was divided by the trapping effort to calculate the number of eggs per day. Thereafter, the mean and standard deviation (sd) from the number of eggs per site and day was calculated. For estimates on the start, end and duration of the active season only sites with a regular occurrence were included to avoid bias caused by individual findings. Thus, only sites where the difference between the first and the last finding of eggs was > 90 days were included.

To investigate possible factors influencing the number of *Aedes*-eggs found in a sample we used the original data to compute a zero-inflated generalized linear mixed model, taking into account the different observation periods. Note that this model has two parts, a Poisson count model (conditional model) and the logit model for predicting excess zeros. However, this analysis was only possible for *Ae. japonicus*, as the sample size in the other *Aedes*-species was too small. In our analysis, we considered the nested structure (positions within sites) with repeated measurements, the zero-inflated structure of the data, as well as differences in the observation period of each sample. The potential influencing factors habitat type, latitude, altitude, daily mean temperature, daily sum of precipitation and daily maximum wind speed were included into the model. Daily weather data were obtained from the Zentralanstalt für Meteorologie und Geodynamik (ZAMG), which provided us with data from the nearest weather station for each site. For the habitat type, we discriminated between “artificial” and “semi-natural and natural” areas. This classification was based on the CORINE Land Cover data (CLC 2018, © Umweltbundesamt & European Union, Copernicus Land Monitoring Service 2018, European Environment Agency (EEA), with funding by the European Union): “artificial” habitats correspond to CLC class “1. Artificial surfaces”, whereas “semi-natural and natural” correspond to “2. Agricultural areas” and “3. Forest and semi natural areas”. For the altitude, which ranged between 113 and 779 m a.s.l., we differentiated between positions at low altitude (≤450 m a.s.l., this value lies approximately in the middle of the observed range in the altitude) and those at high altitude (>450 m a.s.l.). In addition to the model parameters we also give the estimated marginal means to better illustrate the effects of the investigated parameters on the number of eggs. Estimated egg numbers by the model are based on both the zero-inflated, as well as the conditional, part of the model. For all presented estimations of egg numbers, the non-focus parameters were held constant with the following values: habitat type—artificial surfaces, latitude—47.5° N, altitude—113–450 m a.s.l., temperature—20 °C, daily sum of precipitation—0 mm, maximum daily wind speed—8 km/h.

All statistical analyses and the creation of the graphics were conducted using the software Program R Version 4.0.5 [31]. For the calculation of the statistical model we used the “glmmTBM” function from the eponymous package V 1.1.1 [32], and for the marginal means the function “ggpredict” from the “ggeffects” package V 1.1.1 [33]. Graphs and maps were created with the packages “ggplot2” V 3.3.3 [34], “ggmap” V 3.3.0 [35], “rgdal” V 1.5.23 [36] and “cowplot” V 1.1.1 [37].

## 3. Results

### 3.1. The Overall Number of Aedes-Eggs

**Figure 2:**
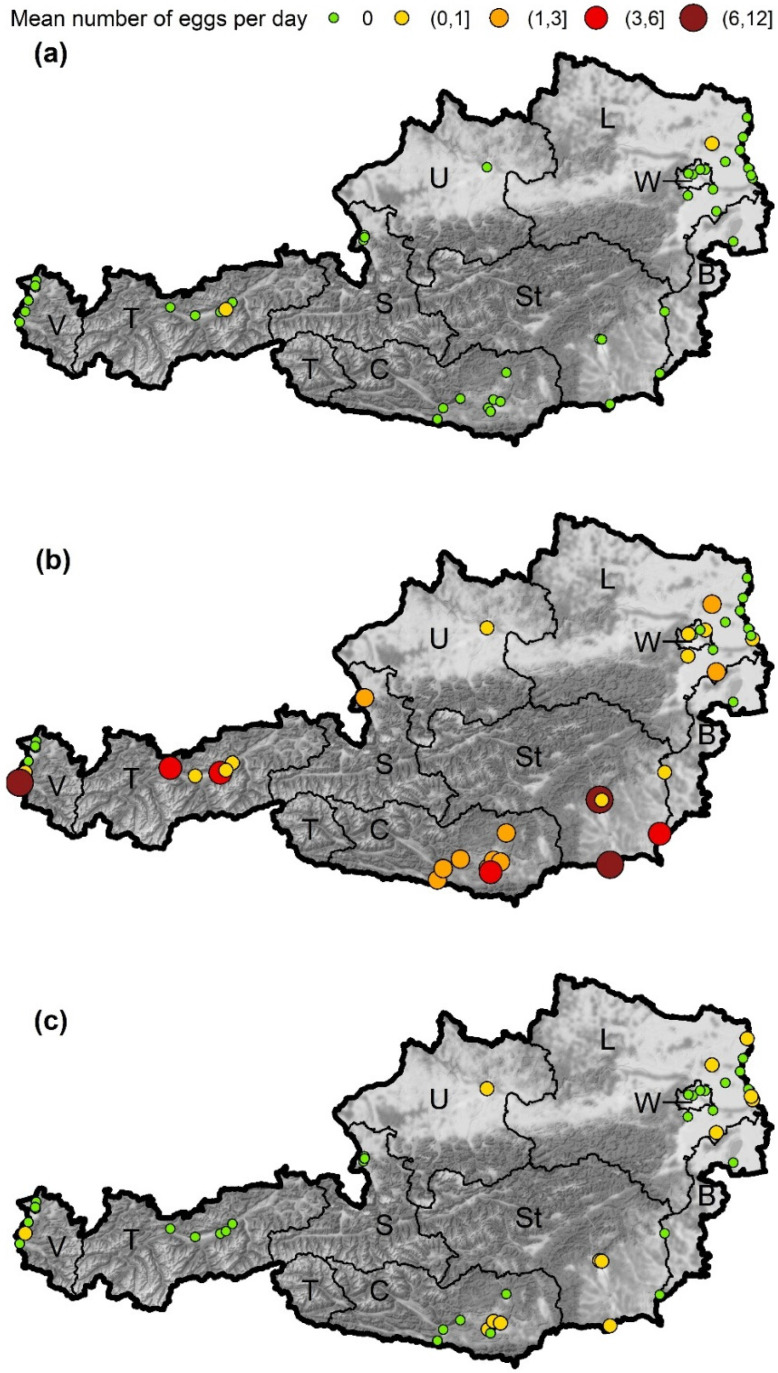
Mean number of eggs per day and site detected in the ovitraps. The number of eggs was categorized, the start and end values given in brackets for values > 0. Round brackets indicate endpoints which are excluded, endpoints with square brackets are included in a category. (**a**) *Ae. albopictus*, (**b**) *Ae. japonicus*, (**c**) *Ae. geniculatus*. Austrian provinces: W—Vienna, L—Lower Austria, B—Burgenland, U—Upper Austria, St—Styria, C—Carinthia, S—Salzburg, T—Tyrol, V—Vorarlberg. Map tiles by Stamen Design, under CC BY 3.0. Data by OpenStreetMap, under ODbL. Data source borders: NUTS units, Statistik Austria—data.statistik.gv.at, accessed on 18 January 2022.

A total of 4521 samples were collected, including 992 (22.0%) containing eggs of container breeding *Aedes* species. In total, 63,287 *Aedes* eggs were counted. *Aedes* eggs were found at a very large number of sites (80.0%, Figure 2, for details see Supplementary Materials). At nine sites no *Aedes* eggs could be detected in the ovitraps: at two sites in Vienna (where one of the sites consisted of only one trap position), the site in Illmitz (Burgenland), three sites in Lower Austria (district Gänserndorf) and at three sites in Vorarlberg. The average number of eggs per day and site was 1.8 ± 6.09. A particularly large number of *Aedes* eggs was found in Styria. In this province at the site in the district of Leibnitz, an average of 11.1 ± 14.96 *Aedes* eggs per position (trap) and sampling day was counted (74.6% of the samples with eggs), and at one of the sites in the city of Graz it was 10.3 ± 11.66 eggs per day (77.7% of the samples with eggs). A large number of eggs was also found in the province of Vorarlberg at the site at the Customs Office Feldkirch Tisis with 10.4 ± 19.89 eggs per day (48.2% of the samples with eggs). We detected eggs from three different container-breeding *Aedes* species within our samples, namely *Ae. albopictus*, *Ae. japonicus* and *Ae. geniculatus*. In addition, as revealed by the genetic analysis, we once found eggs of *Ae. vexans*. However, as this mosquito species breeds in inundation areas and is not a tree-hole-/container-breeding species, it was not included in the analysis. Further eggs on the samples were identified by genetic analysis as Dasyhelea flavifrons (Ceratopogonidae), Helophilus latifrons (Syrphidae) and Clogmia albipunctata (Psychodidae).

### 3.2. Aedes albopictus

Eggs from *Ae. albopictus* could only be detected at two sites (Figure 2a). At the site in Weer (Tyrol, 726 m a.s.l.) a single egg was found at the end of June. The second site was at the motorway A5 rest stop Hochleithen (Lower Austria, 248 m a.s.l.), where four samples with *Ae. albopictus* eggs were found from early July to mid-September. This is the first detection of Asian tiger mosquitoes in the province of Lower Austria.

### 3.3. Aedes japonicus

*Aedes japonicus* was present at all but one sites where *Aedes* eggs were found (Figure 2b). This species was detected in 765 samples, which corresponds to 98.3% of the samples in which a species could be determined by molecular methods. There were large differences in the regional and seasonal occurrence of *Ae. japonicus* eggs. At 23 sites, including all sites in Carinthia (southernmost province) and Styria (south-easternmost province), eggs were found regularly (i.e., >90 days). At eight sites eggs of this species were found less frequently (<90 days) and at 14 sites no *Ae. japonicus* eggs could be detected.

The first eggs of *Ae. japonicus* were detected on 1 May 2020 (Styria), the last eggs on 29 October 2020 (Styria). At sites with a regular occurrence of *Ae. japonicus* the egg-laying season started on average on 29 May 2020 (±20.5 days, *n* = 23) and ended on average on 10 October 2020 (±12.0 days, *n* = 23). Thus, in Austria the mean length of the active season for *Ae. japonicus* was 134 ± 22.0 days (*n* = 23) in 2020. The peak of the season, with the highest mean number of eggs per day (32.7 ± 25.8, *n* = 23) was at the beginning of August (6 August 2020 ± 33.5 days, *n* = 23). The lowest temperature at which *Ae. japonicus* eggs were found was 7.6 °C (average mean daily temperature during the trapping event from 13 to 27 October 2020, Tyrol). We found eggs from *Ae. japonicus* at altitudes ranging from 132 to 779 m a.s.l.

**Figure 3:**
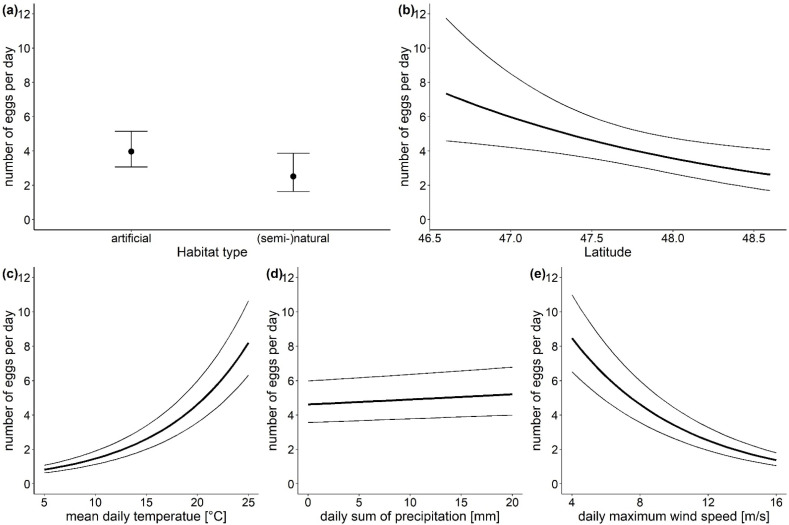
Points and bold lines show the effects of the investigated parameters (**a**) habitat type, (**b**) latitude, (**c**) mean daily temperature, (**d**) daily sum of precipitation and (**e**) daily maximum wind speed on the number of *Ae. japonicus* eggs as predicted by the generalized linear mixed model for count data, error bars and thin lines represent 95% CI. Conditions for non-focus parameters are set to: habitat type—artificial surfaces, latitude—47.5° N, altitude—100–450 m a.s.l., temperature—20 °C, precipitation—0 mm, max. wind speed—8 km/h.

The statistical analysis revealed that the location parameters habitat type and latitude had a strong effect on the number of *Ae. japonicus* eggs found (Table 1). Although the habitat type did not affect whether eggs were laid in an ovitrap, the model results showed, that if eggs were laid, significantly more eggs were found in traps located in artificial habitats (model estimates: 3.97 eggs per day, 95% CI (3.06, 5.15)) than in traps in natural or semi-natural habitats (model estimates: 2.51 eggs per day, 95% CI (1.64, 3.87)) (Figure 3a). Further, the number of eggs decreased with increasing latitude (Figure 3b). For example, in the capital city Vienna (48.2083° N) the model predicts 3.21 eggs per day (95% CI (2.30, 4.47)), while in Klagenfurt, the capital town of the southernmost province Carinthia (46.6167° N) 7.29 eggs per day (95% CI (4.58, 11.59)) are expected in an ovitrap. The three investigated climatic parameters also influenced the number of *Ae. japonicus* eggs (Table 1). With increasing daily mean temperature and precipitation, the number of eggs increased. Although both factors had a significant effect, the effect size for temperature (Figure 3c) was very large, as the estimates from the model showed an increase from 1.46 eggs per day (95% CI (1.13, 1.90)) at 10 °C to 8.21 eggs per day (95% CI (6.33, 10.65)) at 25 °C. In contrast, the effect size for the daily sum of precipitation (Figure 3d) was rather small. The daily maximum wind speed had a strong negative effect on the number of eggs found in an ovitrap (Figure 3e). At wind speeds of 5 km/h 7.28 eggs per day (95% CI (5.61, 9.44)) are estimated by the model for an ovitrap, while at wind speeds of 15 km/h only 1.60 eggs per day (95% CI (1.23, 2.09)) are expected.

### 3.4. Aedes geniculatus

In addition to the investigated AIM species, the native species *Ae. geniculatus* was detected sporadically as well. In 24 samples, eggs of this species were found, in 13 of them together with *Ae. japonicus* eggs. Most findings come from Styria (nine samples with eggs) and Lower Austria (eight samples with eggs), but it has also been found in Carinthia, Vorarlberg, and Upper Austria (Figure 2c). The first *Ae. geniculatus* eggs were documented on 18 June 2020 (Lower Austria), the last on 27 September 2020 (Styria). Samples positive for *Ae. geniculatus* were found at altitudes ranging from 132 to 510 m a.s.l.

## 4. Discussion

The results of this study allow (for the first time) a countrywide overview of the situation of potentially invasive *Aedes* species in Austria using a standardized method. The species of greatest concern, *Ae. albopictus*, was only found at two locations. Although it is problematic to directly compare the results of this study with previous ones, as they used a different sampling-protocol, the single egg found in western Austria (Tyrol) was well below the expected numbers, as this species has been regularly reported in previous years for this area [12,15]. The previous findings of *Ae. albopictus* in this area seems to be the result of (possibly repeated) introductions via road traffic from Italy, where this species is well established [38], which is the most relevant way in which this species is introduced into new areas within the European continent [3,4,39,40,41]. In 2020, however, cross-border road traffic was reduced due to COVID-19 regulations, likely affecting the spread of AIMs during this period. Especially the traffic between Austria and Italy was severely restricted [42], which could explain the unexpected almost-absent *Ae. albopictus* situation in this area. Although travel at the country border was temporarily restricted throughout Austria, the borders to the east were less affected. The first evidence of *Ae. albopictus* in the province of Lower Austria, as documented by this study, is probably the result of an introduction from the Czech Republic, where *Ae. albopictus* has been present near the border to Austria since 2012 [43,44]. However, introduction by road traffic from more distant areas of origin is also possible. At this location at the motorway A5 rest stop Hochleithen eggs were found over a time period of three months, indicating local reproduction of *Ae. albopictus*. In the same year in August, *Ae. albopictus* was also detected by citizens for the first time in the city of Vienna [16], approximately 30 km away from the motorway rest stop Hochleithen. Thus, the location at Hochleithen is a likely stopover documenting from where the introduction of this mosquito species into Vienna occurred.

The results of this study show that nine years after its first introduction, *Ae. japonicus* is well established in Austria. Eggs were found at higher frequencies in the southern parts of Austria, where the establishment of this species in Austria had its origin (in Styria [13]). Previous studies documented that in Austria this species rapidly expanded its range northwards from this origin in the following years and a second, most likely independent, introduction event occurred in western Austria in 2015 [14,27]. The lower abundances in the northern regions of Austria indicate that *Ae. japonicus* may not yet have established stable populations in that area. If this is the case, the effect of latitude on the number of *Ae. japonicus* eggs found in ovitraps should diminish within the next years.

In this study, we found more *Ae. japonicus* eggs in habitats with artificial surfaces (i.e., urban or industrial/transport sites) than in natural and semi-natural areas, which, in our study, were mostly agricultural areas. This is consistent with previous studies from their native range as well as in areas, where this species has been introduced, showing that *Ae. japonicus* are often encountered in urban areas [45,46,47]. Although they prefer natural habitats to artificial ones, preferably colonizing forested areas, especially forest edges, they have been shown to avoid agricultural areas [48,49].

The weather conditions had a strong impact on the number of *Ae. japonicus* eggs found during the study period, especially the temperature. The lowest temperature at which we observed eggs was 7.6 °C. Although this is no precise measurement of the temperature at the time of egg-laying (as it was the average daily temperature during the sampling period), this value fits in with the results from a study from France (Alsace), where the authors conclude that *Ae. japonicus* eggs are laid when temperatures are above 7.5 °C [50] and a laboratory study, estimating that threshold temperature for larval developmental is 7 °C [51]. Our results of an increased number of eggs with increasing temperatures concur with previous field studies [48,50]. We found no decrease in egg numbers at the highest observed temperatures, suggesting that the upper thermal limit of this species [51,52] has, at least on the population level, no relevance to the seasonal dynamics of this species in Austria.

The maximum wind speed had a similar effect size compared to temperature, but in the opposite direction. High wind speeds have been shown to decrease adult mosquito catch rates [53,54,55]. While strong winds could impair flight activity directly, experiments by Hoffmann [56] suggest that a high wind speed impairs the orientation of the mosquitoes by deluding attracting stimuli like CO_2_. Both possibilities, which are not mutually exclusive, would explain the decreased number of eggs at high wind speed. Lower flight activity as well as impaired orientation for finding a host or a suitable breeding habitat would result in a lower number of eggs in the ovitraps.

The number of *Ae. japonicus* eggs increased with the amount of precipitation during the sampling period, even though the effect size was marginal. Positive effects of precipitation on *Ae. japonicus* populations have been observed before, as rainfall in the months preceding the capture event increases the number of breeding sites [57]. However, as rainfall in the days prior to the ovitrap sampling events would increase the number of alternative breeding sites and could also decrease flight activity (as shown for *Culex* mosquitoes [58,59]), the reasons for the observed short-term positive effects still need to be explored.

*Aedes koreicus* has already been found in Austria, but it is very rare and has only been detected at seven occasions until 2020: at two sites in Tyrol (districts Schwarz and Kufstein), at two sites in Western Carinthia (district Hermagor), and at one site in Southern Styria (district Leibnitz) [15,17]. Due to the current rareness of this species, it is not surprising that we did not find *Ae. koreicus* during the monitoring project.

The native tree-hole breeding species *Ae. geniculatus* was not a focus species in this study. The preferred habitat of this species are deciduous forests [48,60,61,62], a habitat not well investigated within our study. However, the presented results will contribute to the knowledge on the distribution of this species in Austria. *Aedes geniculatus* is anthropophilic and a potential vector for *Dirofilaria immitis* and *Dirofilaria repens* and, at least under laboratory conditions, for Chikungunya virus [63,64]. Thus, knowing the distribution of this species in Austria will help to evaluate the outbreak risk of diseases caused by those pathogens better.

With the 45 sites sampled in this study we obtained a first overview of the situation of AIMs in Austria. However, we could not cover the entire country. For example, our monitoring missed the first occurrence of *Ae. albopictus* in Vienna [16]. Thus, to establish an Austrian-wide early warning system for the detection of AIM species, the number of sampling sites should be increased in the future. In addition, a combination of a nationwide monitoring project together with a citizen science project such as “Mosquito Alert” (pan-European) or “Mückenatlas” (Germany), which provide simple tools for citizens to report (alien) mosquito species, would increase the chances for an early detection of AIM species [65,66,67].

Ovitraps are a cheap and simple tool for the detection of AIMs and can easily be handled, even by non-professionals. Therefore, they are well suited to be included in citizen science projects for the detection of AIM species. One of the benefits of the Citizen Science approach is that it reduces travel time for researchers and allows a greater coverage. The time required for the people conducting the controls was low, they stated a time expenditure of one hour per site (5 traps) per week on average. At some sites, however, the volunteers were not able to comply with the recommended sampling interval of one week. Control intervals of two weeks or longer often caused mold formation on the samples, impairing the counting of the eggs as well as species determination. The padded envelope used to send the samples per mail to the laboratory was enough protection for the samples as (in most cases) they arrived undamaged at the lab. However, the padding was necessary, as otherwise many eggs arrived crushed, which made the morphological as well as the genetic species determination difficult or impossible.

Within the framework of this study, it was possible to record the status of AIM species throughout Austria for the first time. We could confirm a known location and detect a potentially new population of *Ae. albopictus* in Lower Austria. Further, now we have an overview of the distribution of *Ae. japonicus* in the country and were able to identify the factors influencing the number of *Ae. japonicus* eggs found at the different sites. In addition, we documented occurrences of the native *Ae. geniculatus* in Austria. All three of those species are potential disease vectors and knowing their distribution will contribute a better estimation of the risk of mosquito-borne disease in Austria. The results of this study will also be a useful baseline for a future documentation of changes in the distribution of those species.

## Figures and Tables

**Table 1:** Model results analyzing the variables influencing the number of *Ae. japonicus* eggs using a zero-inflated generalized linear mixed model, taking into account the different observation periods. Note that this model has two parts, a Poisson count model (conditional model) and the logit model for predicting excess zeros. Number of observations: 4258, within 219 groups position:site and 43 groups within factor site.

Conditional Model	Estimate	Standard Error	*z* Value	*p*
intercept	24.465	9.1326	2.679	0.0074
habitat type—artificial surfaces	0.457	0.2245	2.037	0.0417
latitude	−0.515	0.1926	−2.675	0.0075
altitude—450–800 m a.s.l	0.073	0.2320	0.316	0.7519
temperature	0.115	0.0018	64.725	<0.0001
precipitation	0.006	0.0016	3.761	0.0002
wind speed	−0.151	0.0043	−35.093	<0.0001
random effects				
	Variance	Std. Dev.		
position:site	0.4589	0.6774		
site	0.1319	0.3632		
**Zero-Inflated Model**	**Estimate**	**Standard Error**	***z* Value**	***p***
intercept	−126.355	37.4811	−3.371	0.0007
habitat type—artificial surfaces	−0.708	0.5001	−1.415	0.1572
latitude	2.853	0.7872	3.625	0.0003
altitude—450–800 m a.s.l	−0.937	1.0420	−0.900	0.3683
temperature	−0.329	0.0184	−17.835	<0.0001
precipitation	−0.055	0.0181	−3.057	0.0022
wind speed	0.119	0.0417	2.850	0.0044
random effects				
	Variance	Std. Dev.		
position:site	0.991	0.9955		
site	5.101	2.2585		

## Data Availability

The data presented in this study are available on request from the corresponding author, excluding weather data, as the copyright for these data is held by Zentralanstalt für Meteorologie und Geodynamik (ZAMG).

## Supplementary Materials

The following are available online at https://www.mdpi.com/article/10.3390/insects13030276/s1, Table S1: Information on the conditions at the trapping sites, sampling details and results regarding the *Aedes*-eggs found at each trap site.
